# Complete blood counts with red blood cell determinants associate with reduced beta‐cell function in seroconverted Swedish TEDDY children

**DOI:** 10.1002/edm2.251

**Published:** 2021-05-03

**Authors:** Falastin Salami, Roy N.Tamura, Helena Elding Larsson, Åke Lernmark, Carina Törn, the TEDDY Study Group

**Affiliations:** ^1^ Department of Clinical Sciences Clinical Research Centre Lund University Skåne University Hospital Malmö Sweden; ^2^ Health Informatics Institute Department of Pediatrics University of South Florida Tampa Florida USA

**Keywords:** autoimmunity, glucose metabolism, HbA1c, red blood cells, type 1 diabetes

## Abstract

**Objectives:**

To investigate whether changes in complete blood count (CBC) in islet autoantibody positive children with increased genetic risk for type 1 diabetes are associated with oral glucose tolerance tests (OGTT) and HbA1c over time.

**Methods:**

The Environmental Determinants of Diabetes in the Young (TEDDY) study follows children with increased risk for type 1 diabetes in the United States, Germany, Sweden and Finland. In the current study, 89 Swedish TEDDY children (median age 8.8 years) positive for one or multiple islet autoantibodies were followed up to 5 (median 2.3) years for CBC, OGTT and HbA1c. A statistical mixed effect model was used to investigate the association between CBC and OGTT or HbA1c.

**Results:**

HbA1c over time increased by the number of autoantibodies (*p *< .001). Reduction in mean corpuscular haemoglobin (MCH) and mean cell volume (MCV) was both associated with an increase in HbA1c (*p *< .001). A reduction in red blood cell (RBC) counts (*p *= .003), haemoglobin (*p *= .002) and haematocrit (*p *= .006) levels was associated with increased fasting glucose. Increased red blood cells, haemoglobin, haematocrit and MCH but decreased levels of red blood cell distribution widths (RDW) were all associated with increased fasting insulin.

**Conclusions:**

The decrease in RBC indices with increasing HbA1c and the decrease in RBC and its parameters with increasing fasting glucose in seroconverted children may reflect an insidious deterioration in glucose metabolism associated with islet beta‐cell autoimmunity.

## Abstract 2

Islet beta cell autoantibody positive children with increased risk for developing type 1 diabetes have reduced red blood cell counts and parameters associated with impaired glucose metabolism.

## 1 INTRODUCTION

Autoimmune diabetes also known as type 1 diabetes is a chronic disease affecting children and young people. The incidence rate of autoimmune diabetes among children is increasing worldwide by 3%–4% a year, while the aetiology and pathogenesis are still not fully understood.^1^, ^2^ The disease is associated with a genetic predisposition of class II HLA‐DR‐DQ risk genotypes, accounting for 30%–50% of the genetic type 1 diabetes risk.^3^, ^4^ The HLA risk genotype together with an unknown environmental trigger(s) is causing islet autoimmunity marked by autoantibodies to pancreatic islet beta‐cell proteins that are related to the loss of beta cells and progression to type 1 diabetes. The autoimmune attack is predicted by one or multiple autoantibodies against the beta‐cell autoantigens glutamic acid decarboxylase (GAD), islet antigen‐2 (IA‐2), insulin and Zn transporter 8 (ZnT8).

The Environmental Determinants of Diabetes in the Young (TEDDY) is a multicentre prospective cohort study that aims to investigate environmental factors that trigger islet autoantibodies and type 1 diabetes.^5^ Children at genetic risk for type 1 diabetes are followed from birth until 15 years of age to identify seroconversion to one or several islet autoantibodies (GADA, IAA or IA‐2A) as the first primary end‐point and diagnosis of type 1 diabetes as the second primary end‐point.^6^ The development of two or more islet autoantibodies is increasing the risk for type 1 diabetes above 70% during childhood or adolescence.^7^, ^8^ Previously, we have shown a change in complete blood count (CBC) primarily in counts of neutrophils and red blood cells or levels of haemoglobin or haematocrit in Swedish TEDDY children with multiple islet autoantibodies.^9^ Multiple islet autoantibodies were associated with a reduction of neutrophil and red blood cell counts as well as reduced levels of haemoglobin and haematocrit.^9^

The possible roles of neutrophils, red blood cells, haemoglobin and haematocrit in the pathogenesis of type 1 diabetes are not understood. In a cross‐sectional study, reduced neutrophil counts in islet autoantibody positive adults with increased genetic risk for type 1 diabetes were associated with a reduced beta‐cell function estimated by both fasting and stimulated C‐peptide.^10^ In the present study, the first aim was to investigate whether changes in CBC over time in Swedish TEDDY children positive for one or more islet autoantibodies were associated with glucose metabolism measures (oral glucose tolerance test (OGTT) and haemoglobin A1c (HbA1c)). Second, as the children in this study are longitudinally followed for CBC and HbA1c over time, we tested whether trajectories of these parameters would distinguish single autoantibody from multiple autoantibody positive children. The third aim was to relate the islet autoantibody status at baseline to the change in CBC over time. The hypothesis was that changes in glucose metabolism (OGTT and HbA1c) tracked over time would affect CBC parameters including leukocytes and RBC measures. As CBC is clinical routine and cellular immune mechanisms in type 1 diabetes are not understood, CBC measurements may prove useful to monitor children at increased risk for type 1 diabetes.

## 2 RESEARCH DESIGN AND METHODS

### 2.1 TEDDY study design

The TEDDY study is a longitudinal prospective study composed of six clinical research centres: three in the United States (Colorado, Georgia and Washington) and three in Europe (Finland, Germany, and Sweden). The primary objective of TEDDY was the identification of environmental exposures that are associated with increased risk of type 1 diabetes in children with high‐risk genetic HLA‐DR‐DQ. For all participants, written informed consents were obtained from a parent or a primary caretaker, separately, prior to genetic screening and if eligible prior to enrolment and participation in the TEDDY follow‐up.^11^ The genotype screening of newborns (0–3 months) from general population (90% eligible) and newborns with first degree relatives (10% eligible) was conducted using either a dried blood spot punch or a small volume whole blood lysate specimen format, as previously published.^12^ Enrolled eligible newborns from the general population or with a first degree relative (FDR) had one of the high‐risk HLA genotypes as presented in Table 1. Detailed study design, eligibility and methods have been previously published.^5^, ^6^, ^11^, ^13^ The current study has been approved by the Regional Ethics Review Board in Lund, Sweden and observed by an External Advisory Board formed by the National Institutes of Health, United States.

**TABLE 1:** Eligible high‐risk HLA genotypes for the enrolment in the TEDDY study

TEDDY Code	Full Genotype	Abbreviation	General population
A	DRB1*04‐DQA1*03:01‐DQB1*03:02/DRB1*03‐DQA1*05:01‐DQB1*02:01	DR3/DR4	Yes
B	DRB1*04‐DQA1*03:01‐DQB1*03:02/DRB1*04‐DQA1*03:01‐DQB1*03:02^1^	DR4/DR4	Yes
C	DRB1*04‐DQA1*03:01‐DQB1*03:02/DRB1*08‐DQA1*04:01‐DQB1*04:02	DR4/DR8	Yes
D	DRB1*03‐DQA1*05:01‐DQB1*02:01/DRB1*03‐DQA1*05:01‐DQB1*02:01	DR3/DR3	Yes
E	DRB1*04‐DQA1*03:01‐DQB1*03:02/DRB1*04‐DQA1*03:01‐DQB1*02:01	DR4/DR4	No
F	DRB1*04‐DQA1*03:01‐DQB1*03:02/DRB1#‐DQA1*01:01‐DQB1*05:01^2^	DR4/DR1	No
G	DRB1*04‐DQA1*03:01‐DQB1*03:02/DRB1*13‐DQA1*01:02‐DQB1*06:04	DR4/DR13	No
H	DRB4*01‐ DQA1*03:01‐DQB1*03:02/ DRB1*04‐DQA1*03:01‐DQB1*03:04	DR4/DR4	No
I	DRB1*04‐DQA1*03:01‐DQB1*03:02/RB1*09‐DQA1*03:01‐DQB1*03:03	DR4/DR9	No
J	DRB1*03‐DQA1*05:01‐DQB1*02:01/DRB1*09‐DQA1*03:01‐DQB1*03:03	DR3/DR9	No

The DR4 subtypes DRB1*0403 were excluded.^1^ Acceptable alleles in this haplotype include both DQB1*0302 and *0304.^2^ In this DQB1*0501 haplotype, DR10 must be excluded. Only DR1 is eligible.

### 2.2 Study population and complete blood count (CBC) measurement

**FIGURE 1:**
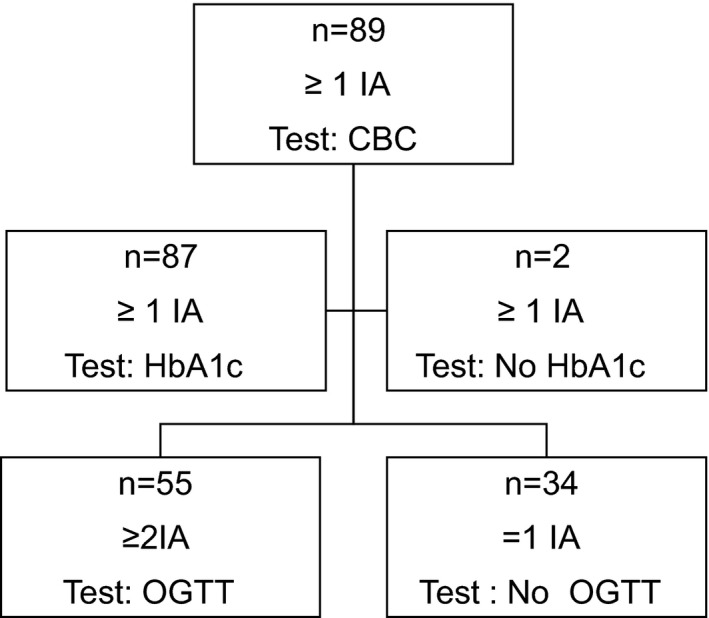
Flow chart of islet autoantibody (IA) positive TEDDY children included in this study and for whom the different tests of CBC, HbA1c and OGTT data were available

The current study included 89 (37 girls and 52 boys), 4–15 years old, Swedish TEDDY children with at least one autoantibody (GADA, IAA, IA‐2A) and if positive for one or more islet autoantibodies, the ZnT8 transporter autoantibody (ZnT8A) was also included (Table 2). The children were followed longitudinally with CBC measurements, analysed at their scheduled TEDDY protocol required follow‐up visits, every third month.^5^, ^11^ The CBC follow‐up was initiated in June 2014 and completed in April 2019. The flow chart in Figure 1 presents the number of islet autoantibody (IA) positive TEDDY children included in this study and for whom the different tests of CBC, HbA1c and OGTT data were obtained. A blood sample was drawn in 8 ml BD Vacutainer**®** CPT**™** tubes supplemented with sodium citrate as anti‐coagulant, from which 300 µl was taken for CBC analysis within 8 h after blood draw. CBC includes cell counts (cells ×10^9^/L) of white blood cells (WBC), neutrophils (Neu), lymphocytes (Lym), monocytes (Mono), eosinophils (EOS), basophils (Baso), platelets (PLT), counts (cells ×10^12^/L) of red blood cells (RBC) and red blood cell indices; haemoglobin (HGB) (g/L), haematocrit (HCT) (L/L), mean corpuscular haemoglobin (MCH) (pg), mean corpuscular haemoglobin concentration (MCHC) (g/L), mean corpuscular volume (MCV) (fL) and red cell distribution width (RDW) (% coefficient of variation). CBC was determined in a multiparameter automated haematology analyser (CELL‐Dyn Ruby; Abbott Laboratories, Diagnostic Division) operated as previously described according to the manufacturer's manual of instructions.^9^, ^14^

**TABLE 2:** Characteristics of all children in the study cohort with one or multiple persistent confirmed islet autoantibodies (IA)

	Persistent confirmed IA *n *= 89	Single IA *n *= 34	Multiple IA *n* = 55
Gender			
Girls	37 (42%)	15 (44%)	22 (40%)
Boys	52 (58%)	19 (56%)	33 (60%)
HLA‐DR/DQ			
DR3‐DQ2/DR4‐DQ8	47 (53%)	17 (50%)	30 (55%)
DR4‐DQ8/DR4‐DQ8	17 (19%)	3 (8%)	14 (25%)
DR4‐DQ8/DR8‐DQ4	14 (16%)	7 (21%)	7 (13%)
DR3‐DQ2/DR3‐DQ2	11 (12%)	7 (21%)	4 (7%)
Number of IA at first CBC			
0	6 (7%)	4 (12%)	2 (4%)
1	35 (39%)	30 (88%)	5 (9%)
2	21 (24%)	0	21 (38%)
3	27 (30%)	0	27 (49%)
Age at first CBC (years)			
Median (SD)	8.8 (1.8)	9.3 (1.5)	8.1 (1.8)
Min‐Max	5.0–12.0	5.2–12.0	5.0–11.4
CBC follow‐up (years)			
Median (SD)	2.3 (1.7)	2.5 (1.8)	2.0 (1.6)
Min‐Max	0.0–4.9	0.0–4.7	0.0–4.9

IA is islet autoantibodies, CBC is complete blood count and SD standard deviation.

### 2.3 Detection of islet autoantibodies

The three different islet autoantibodies (GADA, IAA and IA‐2A) were analysed in two reference laboratories, in the United States at Barbara Davis Center for Childhood Diabetes at the University of Colorado Denver and in Europe at the University of Bristol, the U.K, by radio binding assays as previously described. These two laboratories have high sensitivity and high specificity as well as concordance.^15^ If positive in one of the two reference laboratories, the sample was confirmed in the other laboratory. ZnT8A was analysed in one of the two reference laboratories and only in subjects positive for at least one of the other three autoantibodies. The islet autoantibody status was determined from three months of age and if positive for one or more islet autoantibodies each third month thereafter until either 15 years of age or the diagnosis of type 1 diabetes. Persistent autoimmunity was defined after confirmed in Denver and Bristol positive islet autoantibodies on at least two consecutive visits.

### 2.4 Oral Glucose Tolerance Test (OGTT)

Once the TEDDY child developed a second islet autoantibody, an OGTT was performed twice a year on a scheduled TEDDY visit. Children with one or no islet autoantibodies were not subjected to OGTT. Based on observations in TrialNet, the Finnish DIPP and the German BABY DIAB studies, children with only one islet autoantibody rarely show a deterioration in the OGTT. This is in contrast to children with two or more autoantibodies which over time also develop Impaired Glucose Tolerance (IGT). TEDDY children with two or more autoantibodies were therefore asked to perform a complete OGTT. The amendment to the TEDDY protocol was also done in consideration of the large blood volume taken from the children when a complete OGTT was done. At the TEDDY clinics in Sweden, the 2‐hour OGTT capillary blood glucose was determined by a glucometer (Hemocue® Glucose 201 system, HemoCue AB). The fasting plasma sample was used to determine glucose, insulin and C‐peptide representing fasting glucose (glucose (0)), glucose (120 min, OGTT), fasting insulin (insulin (0)) and fasting C‐peptide (C‐peptide (0)). Glucose, C‐peptide and insulin levels were analysed at The Northwest Lipid Metabolism and Diabetes Research Laboratories, University of Washington, Seattle, WA. Glucose levels were determined using the recognized hexokinase method.^16^, ^17^ Fasting insulin and C‐peptide levels were determined using Tosoh reagents on TOSOH 2000 autoanalyzer (TOSOH, Biosciences, Inc.) as described.^18^

### 2.5 Haemoglobin A1c (HbA1c)

Samples for HbA1c analysis were obtained at every scheduled visit in TEDDY children positive for at least one islet autoantibody. The HbA1c sample was processed and analysed at the Diabetes Diagnostic Laboratory (DDL), University of Missouri as described.^19^, ^20^

## 3 STATISTICAL METHODS

### 3.1 HbA1c, CBC and number of islet autoantibodies

CBC data from subjects with at least one persistent confirmed autoantibody were analysed by mixed model repeated measures analysis similar to our previous report.^9^ Specifically, a CBC end‐point (e.g., white blood cell count) was the dependent variable and independent fixed variables for age, gender, HLA category and time dependent variables of number of positive islet autoantibodies and HbA1c. A random intercept and slope for age for each subject was assumed with unstructured covariance for the random effects. HbA1c was also analysed in a similar mixed model repeated measures analysis with the number of positive islet autoantibodies as the sole time dependent variable. Best linear unbiased predictions^21^ for each subject were estimated for HbA1c and CBC’s of interest from the mixed model analyses.

### 3.2 CBC association with OGTT measures

Subjects with multiple persistent confirmed autoantibodies had their OGTT data summarized by four measures, glucose at time 0 (fasting), glucose at time 120 min and insulin at time 0. CBC data from subjects with multiple persistent confirmed autoantibodies were analysed with mixed model repeated measures analysis with each CBC end‐point as the dependent variable. Independent variables included fixed variables for age, gender and HLA category; the intercept and slope for age were assumed to be random. Because of the high correlation across the four OGTT measures, the best subset of OGTT measures with a cut‐off level of 0.05 was used for analysis and reporting.

## 4 RESULTS

### 4.1 CBC association with HbA1c

The longitudinal HbA1c and CBC measurements taken during a median period of 2.3 (SD 1.7) years and a maximum period of 4.9 years from children with one or multiple persistent confirmed islet autoantibodies are summarized in Table S1. A total of 89 islet autoantibody positive children were followed quarterly with CBC and 87 of them with HbA1c. The association between CBC and HbA1c is shown by mixed model repeated measures results (Table 3). As known, counts of white blood cells, lymphocytes, eosinophils, basophils, platelets, red blood cells and levels of haemoglobin, haematocrit, MCHC and MCV are all age‐dependent. Consistent with our earlier findings, white blood cell counts were significantly (*p *< .001) associated with the number of islet autoantibodies and this association was primarily due to the same significant (*p *< .001) association of reduced neutrophil counts. Counts of lymphocytes (*p *= .018) and monocytes (*p *= .050) also tended to be associated with the number of islet autoantibodies. HbA1c levels increased by decreasing levels of both MCH (Estimate (SE) = −1.51 (0.3)) (*p *< .001) and MCV (Estimate (SE) = −3.67 (0.57)) (*p *< .001). This association was affected by the four type 1 diabetes highest‐risk HLA‐DR‐DQ genotypes (DR3‐DQ2/DR4‐DQ8, DR4‐DQ8/DR4‐DQ8, DR4‐DQ8/DR8‐DQ4 and DR3‐DQ2/DR3‐DQ2) and either IAA first or GADA first for both MCH (*p *= .019, *p *< .001) and MCV (*p *< .001, *p *= .004) (Figure S2).

**TABLE 3:** Association between complete blood count (CBC) and HbA1c, age or gender presented as mixed model regression parameter estimates for 87 islet beta‐cell autoantibody positive children. P‐values for the association between CBC and number of autoantibodies are also shown.

CBC	HbA1c estimate (SE)	Age estimate (SE)	Female estimate (SE)	Number of autoantibodies p‐value
White blood cells	0.73 (0.32) *p*=0.249	−0.10 (0.04) ***p*=0.010**	0.38 (0.19) *p*=0.054	**<0.001**
Neutrophils	0.08 (0.22) *p*=0.711	0.00 (0.02) *p*=0.901	0.22 (0.11) *p*=0.057	**<0.001**
Lymphocytes	0.23 (0.12) *p*=0.055	−0.09 (0.01) ***p*<0.001**	0.17 (0.09) ***p*=0.049**	**0.018**
Monocytes	0.04 (0.03) *p*=0.249	−0.01 (0.00) *p*=0.132	−0.00 (0.02) *p*=0.915	**0.050**
Eosinophils	0.03 (0.05) *p*=0.587	−0.02 (0.00) ***p*=0.015**	−0.02 (0.04) *p*=0.552	0.871
Basophils	0.00 (0.00) *p*=0.133	−0.00 (0.00) ***p*=0.013**	−0.00 (0.00) *p*=0.877	0.689
Platelets	24.80 (12.60) ***p*=0.050**	−10.40 (1.50) ***p*<0.001**	11.70 (8.70) 0.181	0.248
Red blood cells	0.26 (0.19) 0.175	0.17 (0.02) **<0.001**	−0.13 (0.11) 0.249	0.452
Haemoglobin	−0.40 (7.10) *p*=0.952	5.00 (0.80) ***p*<0.001**	−3.00 (5.10) 9=0.564	0.369
Haematocrit	0.00 (0.01) *p*=0.938	0.01 (0.00) ***p*<0.001**	−0.00 (0.00) *p*=0.540	0.452
MCH	−1.51 (0.30) ***p*<0.001**	−0.02 (0.04) *p*=0.540	0.31 (0.26) *p*=0.228	0.074
MCHC	−5.40 (3.00) *p*=0.070	−0.90 (0.30) ***p*=0.007**	−1.40 (1.70) *p*=0.409	0.292
MCV	−3.67 (0.57) ***p*<0.001**	0.15 (0.07) ***p*=0.041**	1.71 (0.58) ***p*=0.002**	0.173
RDW	0.08 (0.17) *p*=0.633	0.02 (0.02) *p*=0.328	−0.05 (0.13) *p*=0.678	0.573

### 4.2 CBC association with OGTT measures

The red blood cell counts, levels of haemoglobin, haematocrit, MCV, MCH and RDW in children with multiple islet autoantibodies were all associated with at least one of the glucose metabolism measures obtained from the OGTT (Table 4). Red blood cell counts, haematocrit and haemoglobin levels in 49 children positive for multiple islet autoantibodies decreased by the increasing levels of fasting glucose and increased by the increase of fasting insulin level. Similar to the RBC, haemoglobin and haematocrit, an increase in MCH was associated with an increase in fasting insulin. RDW was associated with an increase in fasting insulin. Increased white blood cell, lymphocyte, neutrophil and basophil counts were all associated with an increase in the glucose (120 min) measure.

**TABLE 4:** Associations between different CBC with OGTT measures in children with multiple autoantibodies.

CBC	n	Glucose (0) estimate (SE)	Glucose (120) estimate (SE)	Insulin (0) estimate (SE)
Red blood cells	49	‐ 0.023 (0.008) *p*=0.003	‐	0.13 (0.03) *p*<0.001
Haematocrit	49	−0.002 (0.0006) *p*=0.002	‐	0.011 (0.002) *p*<0.001
Haemoglobin	49	−0.58 (0.20) *p*=0.006	‐	3.9 (0.8) *p*<0.001
MCV	50	‐	‐	‐
MCH	54	‐	‐	0.13 (0.05) *p*=0.021
RDW	54	‐	‐	−0.55 (0.024) *p*=0.021
White blood cells	50	‐	0.007 (0.002) *p*=0.002	
Neutrophils	50		0.004 (0.002) *p*=0.016	
Lymphocytes	50		0.003 (0.001) *p*=0.008	
Basophils	50		0.00008 (0.00004) *p*=0.021	

Glucose (0); fasting glucose, Insulin (0); fasting insulin.

### 4.3 Trajectories of neutrophils and HbA1c

**FIGURE 2:**
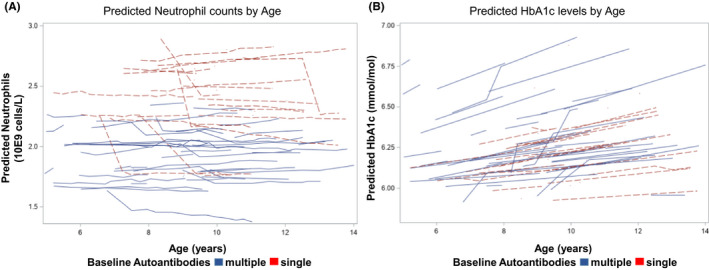
Predicted trajectories from the mixed models analysis for neutrophils (A) and HbA1c (B) stratified by the number of islet autoantibodies (single or multiple ≥2 islet autoantibodies) at initial observation. Each trajectory represents a single subject

The mixed model analysis of HbA1c as the dependent variable indicated that the number of islet autoantibodies was associated with increased levels of HbA1c (*p *< .001). The increase was primarily due to the cohort with three islet autoantibodies when compared to the cohort with two islet autoantibodies, the estimated increase was 0.17 (SE = 0.04, *p *< .001). Since the study population was longitudinally followed for CBC and HbA1c, predicted trajectories over the 2–4.9 years of follow‐up for each subject were estimated from the linear mixed model analysis. Each subject was categorized by their initial baseline autoantibody status (single or multiple ≥2 islet autoantibodies) to investigate any difference between children with single or multiple islet autoantibodies. No difference could be found in predicted trajectories for monocytes, lymphocytes, red blood cells, haemoglobin, MCH and MCV (Figure S1). A difference was identified in predicted trajectories for both neutrophils and HbA1c where the number of islet autoantibodies had a significant impact (*p *< .001) on the predicted values (Figure 2). Predicted trajectories for neutrophils resulted in a decrease of neutrophil counts in children with multiple islet autoantibodies compared to children with a single islet autoantibody. The subjects with multiple islet autoantibodies at baseline had lower predicted neutrophil counts than those with single islet autoantibody at baseline, and also, those single autoantibody positive subjects had large reductions in predicted values of neutrophils when the number of islet autoantibodies increased (Figure 2A). Age seemed to have little effect on the predicted neutrophil counts (linear age *p* = 0.901). In contrast, predicted values of HbA1c increased by both age (*p *< .001) and the number of islet autoantibodies (*p *< .001). Variability in the rate of increase of HbA1c, estimated by the slopes in Figure 2B, appears to be low across these children.

## 5 DISCUSSION

CBC is one of the most important and commonly used clinical laboratory tests. It gives the differential of white blood cells, red blood cell counts, haemoglobin levels and red blood cell indices and thus provides information about the immune system, the production of all blood cells and identifies the oxygen‐carrying capacity.^22^ CBC is not well studied in children at increased genetic risk for type 1 diabetes and positive for one or multiple islet autoantibodies of GADA, IAA, IA‐2A or ZnT8A. Consistent with our previous CBC study in Swedish TEDDY children, the number of autoantibodies was significantly associated with changes in neutrophil and lymphocyte counts.^9^ This is expected since the 72 islet autoantibody positive children included in the previous study have also continued to be followed up in the current study for a longer period and account for 80% of the children included. Interestingly, results from the current study revealed that these changes in leukocyte counts were not associated with glucose metabolism. The major findings in this study were the significant associations in children with multiple islet autoantibodies between the red blood cell counts, parameters, and indices with glucose metabolism, estimated with fasting glucose, fasting insulin and HbA1c as products of beta‐cell function.

The strength in our study is the longitudinal follow‐up of 89 seroconverted children at increased risk for type 1 diabetes during almost five years of prospective CBC, OGTT and HbA1c measurements.

Some subjects with type 1 diabetes do have a peripheral insulin resistance that causes the plasma glucose levels to remain at increased levels for a longer time upon a glucose challenge.^23^ The decrease of MCV and MCH with increased levels of HbA1c, the decrease of red blood cell counts, levels of haemoglobin and haematocrit with the increase in fasting glucose are all indicating a disorder of the red blood cell homeostasis and function associated with increased blood glucose levels or impaired glucose metabolism. The increase in red blood cell counts, levels of haemoglobin, haematocrit, MCH and the decrease of RDW reflecting a normal blood cell homeostasis was associated with an increase in fasting insulin reflecting a normal beta‐cell function. However, increased fasting insulin in children with multiple autoantibodies is a sign of beta‐cell destruction or leakage of insulin.^24^ These new findings have to our knowledge not been presented before and may suggest an impaired haematopoietic cell production in the bone marrow related to the pathogenesis of type 1 diabetes due to loss of beta‐cell function, destruction or both.

A potential weakness of the study is that only Swedish children from one of the three Swedish TEDDY clinics were followed up for CBC, which resulted in a smaller cohort. Lack of resources was one reason why CBC was not carried out at other TEDDY sites. For the same reason, ferritin levels have not been measured routinely in TEDDY.

While MCV defines the size, MCH defines the haemoglobin mass of the red blood cell and a decrease in those indicates the senescence of red blood cells.^25^ Low levels of haemoglobin, MCV and MCH usually reflect a deficient storage of iron due to low intake from the diet or low levels of ferritin.^26^ Further studies are needed to shed light on this issue in healthy islet autoantibody positive subjects. The significant inverse association between HbA1c with MCV and MCH as presented in this study is important for two reasons: the first is that a relative decrease in insulin may affect erythropoietin or other factors that are important to erythropoiesis, and the second is that MCV and MCH could be useful for better prediction of the time to type 1 diabetes in children with multiple islet autoantibodies. It is well known that insulin is a general regulator of protein synthesis; therefore, it might be speculated that innate deterioration of beta‐cell function may be the reason for the relationships observed. Therefore, a primary decrement in the level of beta‐cell function could be leading to lower levels of insulin secretion, which could be leading to a decrease in haemoglobin synthesis. Besides, experimental studies have found that erythropoietin decreases the circulating levels of glucose.^27^ Therefore, it might be speculated that a diminished erythropoiesis due to low levels of erythropoietin may be an underlying factor for diminished glucose metabolism that cause increased levels of glucose and HbA1c.

The pathogenic process of type 1 diabetes is heterogeneous with differences in the initiation of islet autoimmunity by either IAA first or GADA first depending on HLA risk genotype and yet to be identified environmental triggers.^28^, ^29^, ^30^, ^31^ This data may explain the association between HLA‐DR‐DQ and the first appearing islet autoantibody (GADA or IAA) and an interaction with the inverse association between HbA1c and MCV or MCH. An association between diabetes risk and MCV and MCH was also shown previously among young adults,^32^ adults^33^ and pre‐menopausal women.^34^ Furthermore, several studies have shown an increase in HbA1c levels with iron‐deficiency that negatively affects the red blood cell indices.^35^ This information together with our results of the inverse association between MCV and MCH with HbA1c represents a process that takes place already during an autoimmune attack on the beta cells and may remain until after diagnosis of type 1 diabetes. Intriguingly, data from non‐diabetic pregnant women have suggested that there is a positive correlation between HbA1c and haemoglobin, haematocrit and MCHC.^36^

Because HbA1c is only tested in seroconverted children in TEDDY, future studies should determine whether this association occurs before or after seroconversion.

The beta‐cell function was investigated through OGTT measurements. A decrease in the red blood cell counts, haematocrit and haemoglobin levels was associated with an increase of fasting blood glucose levels that reflects the progression to impaired glucose tolerance (stage 2 in type 1 diabetes staging).^37^ In contrast, the increase in red blood cell counts, haemoglobin, haematocrit and MCH levels and the decrease in RDW were all associated with an increase of fasting insulin level, reflecting a normal beta‐cell function and a normal red blood cell status. The increase of fasting insulin in children with multiple autoantibody may also reflect beta‐cell dysfunction or damage leading to insulin leakage.^24^

Our hypothesis indicated above is that the conspicuous loss of beta‐cell function leading to increased blood glucose levels would cause bone marrow failure affecting the erythropoiesis and thereby red blood cells and their parameters. Slowly rising HbA1c and a slowly deteriorating glucose metabolism over time within normal limit values may affect the erythropoiesis. The insidious reduction over time needs to be explained. It may be due to a slowly diminishing insulin production which may affect erythropoiesis. An alternative is that an insidious increase in HbA1c is detrimental also to early steps in the erythropoiesis.^38^

The increase in white blood cell counts primarily by the increase of lymphocytes followed by the neutrophils and basophils was associated with increased 120 minute time point glucose indicating impaired glucose tolerance. Earlier studies in both healthy and diabetic adults have shown elevated white blood cell counts associated with impaired glucose tolerance suggesting the white blood cell count as a measure of risk for impaired glucose metabolism.^39^, ^40^

The novel finding presented by the predicted trajectories of neutrophil counts is that the counts are stable by age and as already shown in previous studies reduced by the number of autoantibodies.^9^, ^41^ The lower predicted neutrophil counts in children with multiple autoantibodies need further investigation as the reduced numbers of neutrophils may contribute to the disease pathogenesis.

As presented from the predicted trajectories, both age and number of autoantibodies are affecting the HbA1c predicted values. However, the effect of the numbers of autoantibodies was detected regardless of the age of the autoantibody positive child. HbA1c has been suggested to predict time to clinical onset of type 1 diabetes in children at risk.^42^, ^43^ Predicted trajectories of HbA1c could therefore be of great importance in the clinic to further develop a model to predict time to clinical onset of type 1 diabetes.

## 6 CONCLUSION

There are negative associations between red blood cell counts, haemoglobin and haematocrit with increased fasting glucose and for the indices (MCH and MCV) also increasing HbA1c indicating a progression to a diminished glucose metabolism and a reduced beta‐cell function in seroconverted children with increased risk for developing type 1 diabetes. In contrast, a positive correlation was found between red blood cell count, haemoglobin, haematocrit, MCH and a negative association for RDW with increasing fasting insulin may indicate a normal beta‐cell production of insulin but also insulin leakage due to beta‐cell damage. The significant negative associations suggest an unknown cellular mechanism that may originate from the early haematopoiesis in the bone marrow, triggered by a more aggressive autoimmune attack on the beta cells resulting in increased blood glucose levels and later a total beta‐cell loss and type 1 diabetes. Subtle and insidious changes in glucose levels may also affect the bone marrow resulting in changes in the red blood cell counts and the levels of its parameters. Further investigation of erythrocytes and the erythropoiesis in children positive for islet autoantibodies may help to better define the stages of the pathogenesis prior to type 1 diabetes clinical diagnosis.

## CONFLICTS OF INTEREST

The authors declare no conflict of interest.

## AUTHOR CONTRIBUTIONS

F.S. was overseeing the CBC analyses, interpreted data and wrote the manuscript. R.T. performed statistical analyses, reviewed and edited the manuscript. H.E.L. and C.T. reviewed and edited the manuscript. Å.L. conceived the study, contributed to study design and reviewed and edited the manuscript. Å.L. is the guarantor of this work and, as such, had full access to all the data in the study and takes responsibility for the integrity of the data and the accuracy of the data analysis.

## Supporting information

Fig S1‐S2

Supplementary Material

## Data Availability

All the generated and analysed data presented in this study will be made available in the National Institute of Diabetes and Digestive and Kidney Diseases (NIDDK) Central Repository at https://www.niddkrepository.org/studies/teddy.
