# Supplementary material for: Complete blood counts with red blood cell determinants associate with reduced beta‐cell function in seroconverted Swedish TEDDY children
Source: Endocrinol Diabetes Metab. 2021 May 3;4(3):e00251. doi: 10.1002/edm2.251 (PMC8279594; doi:10.1002/edm2.251)
Supplement: Supplementary file 1 — Fig S1‐S2 [file EDM2-4-e00251-s001.pptx]

## Slide 1
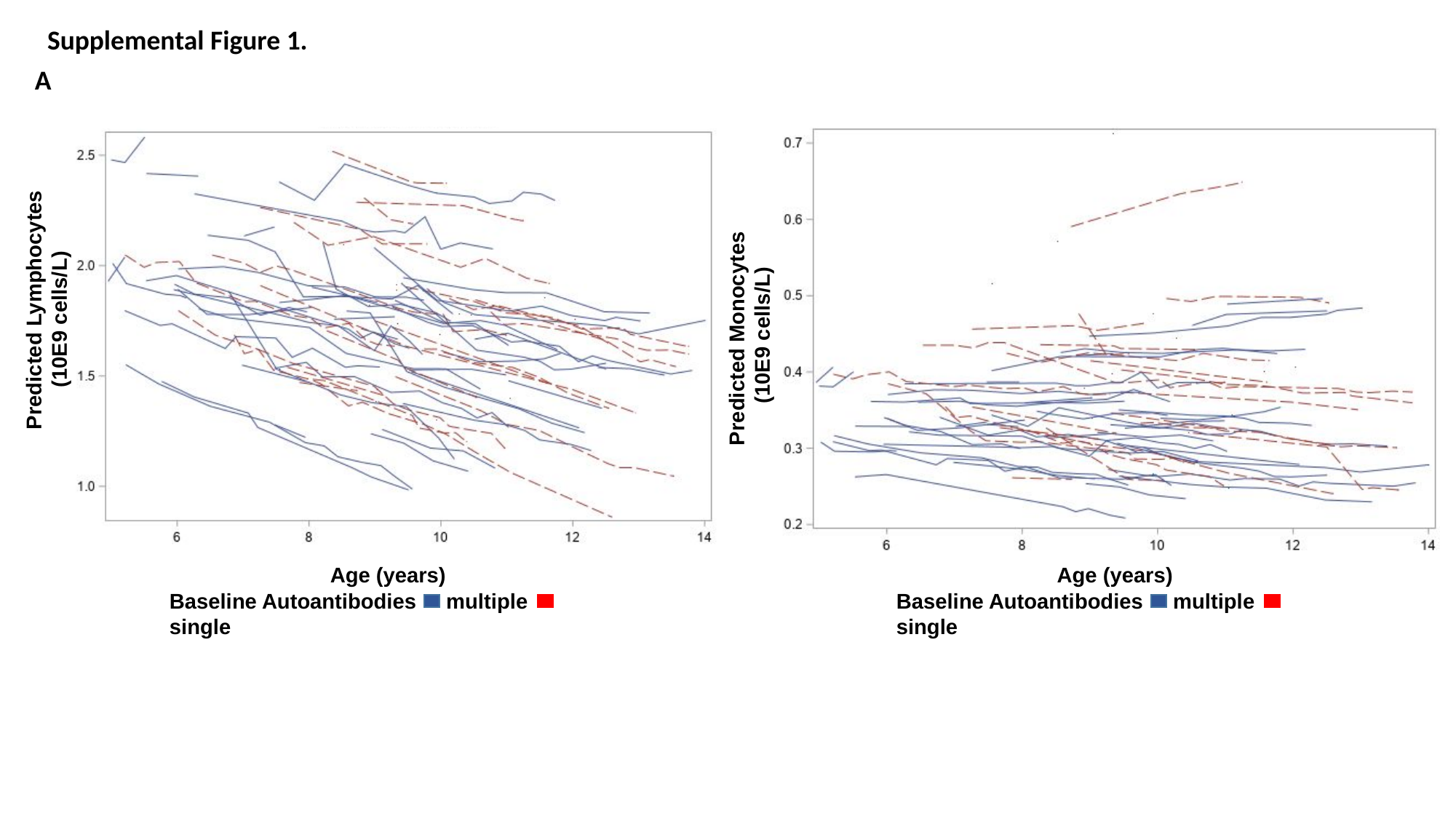

Supplemental Figure 1.
A
Predicted Lymphocytes
 (10E9 cells/L)
Predicted Monocytes
 (10E9 cells/L)
 Age (years)
Baseline Autoantibodies multiple single
 Age (years)
Baseline Autoantibodies multiple single

## Slide 2
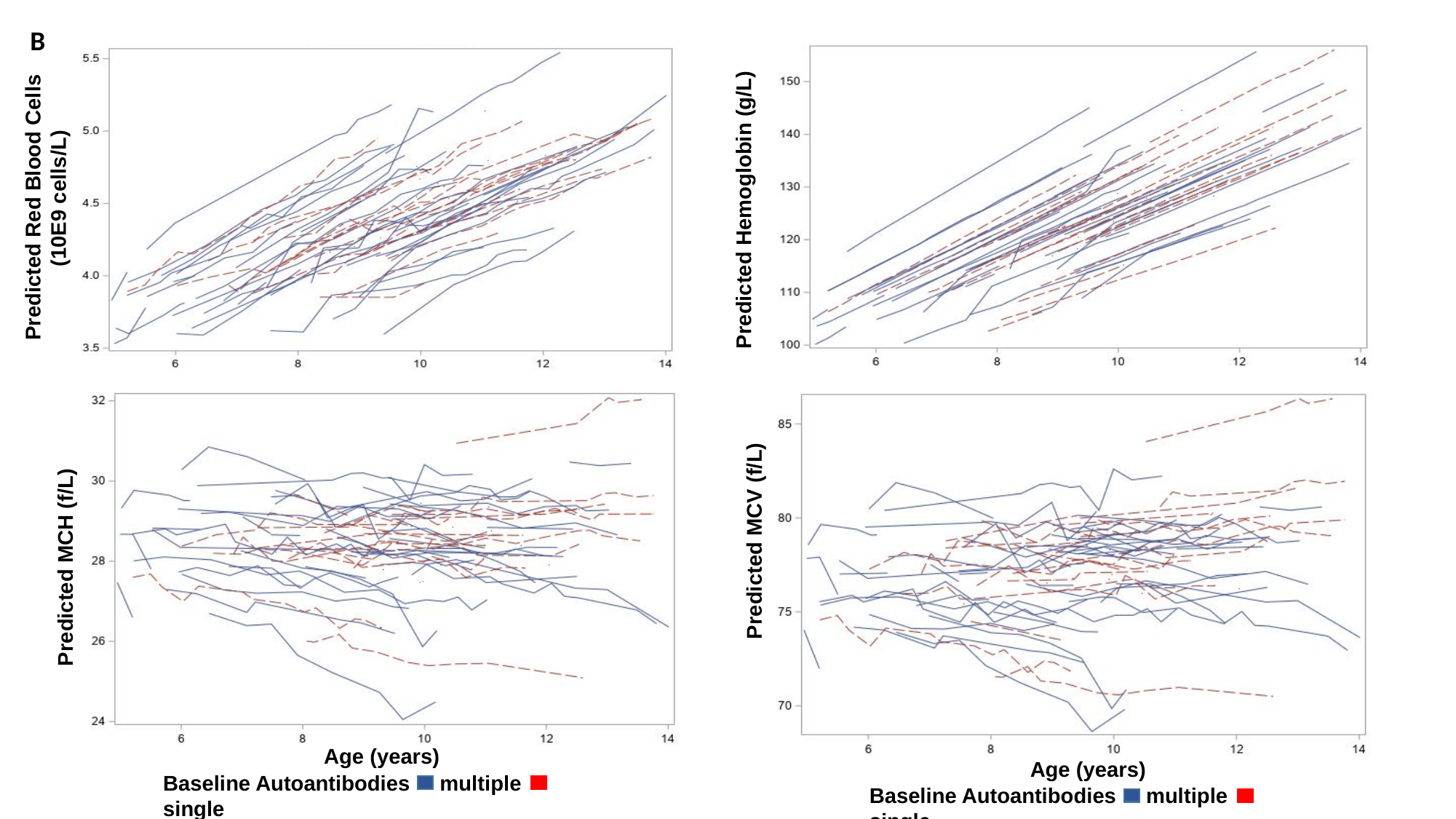

B
Predicted Red Blood Cells (10E9 cells/L)
 Predicted Hemoglobin (g/L)
 Predicted MCV (f/L)
 Predicted MCH (f/L)
 Age (years)
Baseline Autoantibodies multiple single
 Age (years)
Baseline Autoantibodies multiple single

## Slide 3
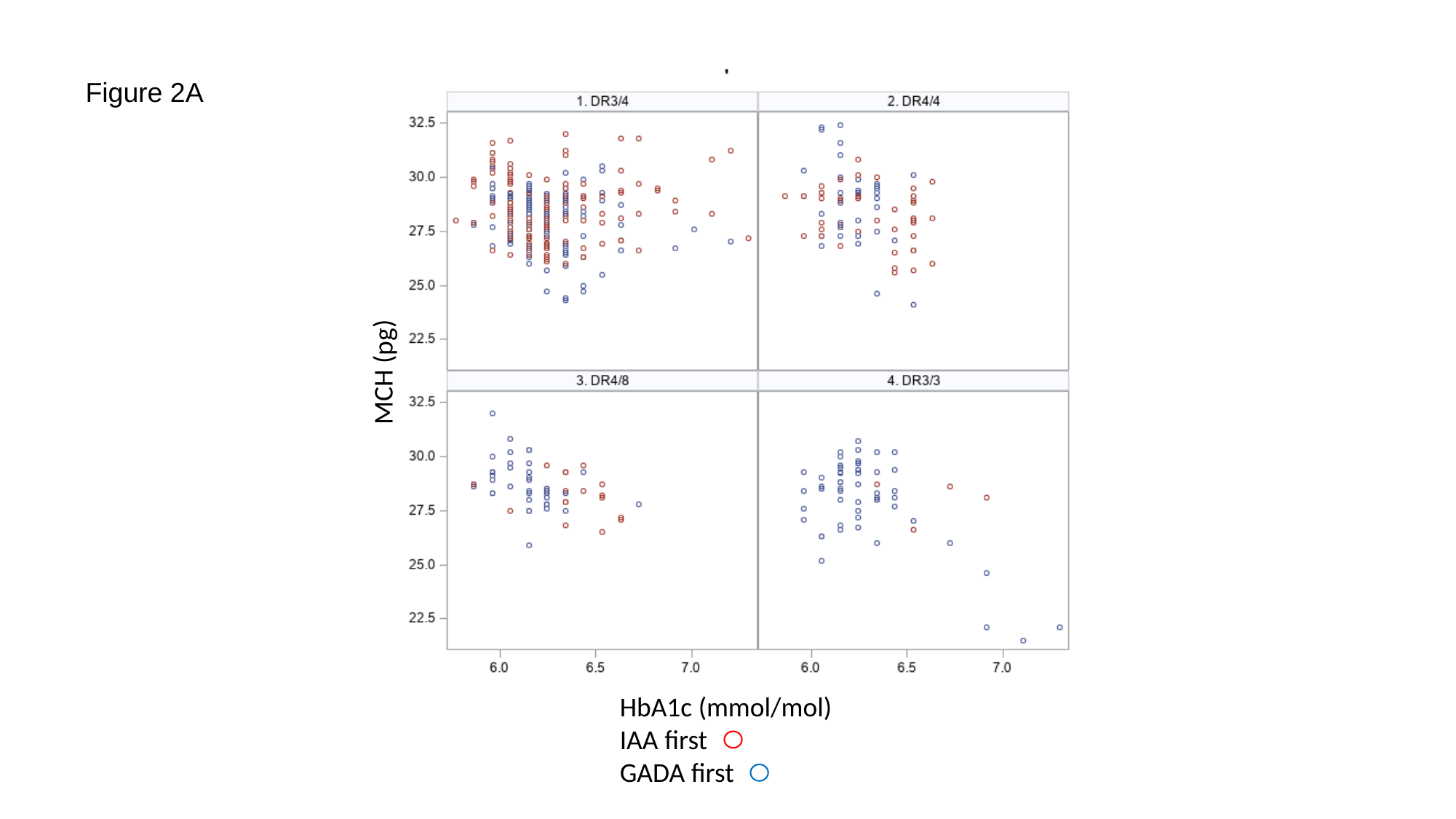

Figure 2A
MCH (pg)
HbA1c (mmol/mol)
IAA first
GADA first

## Slide 4
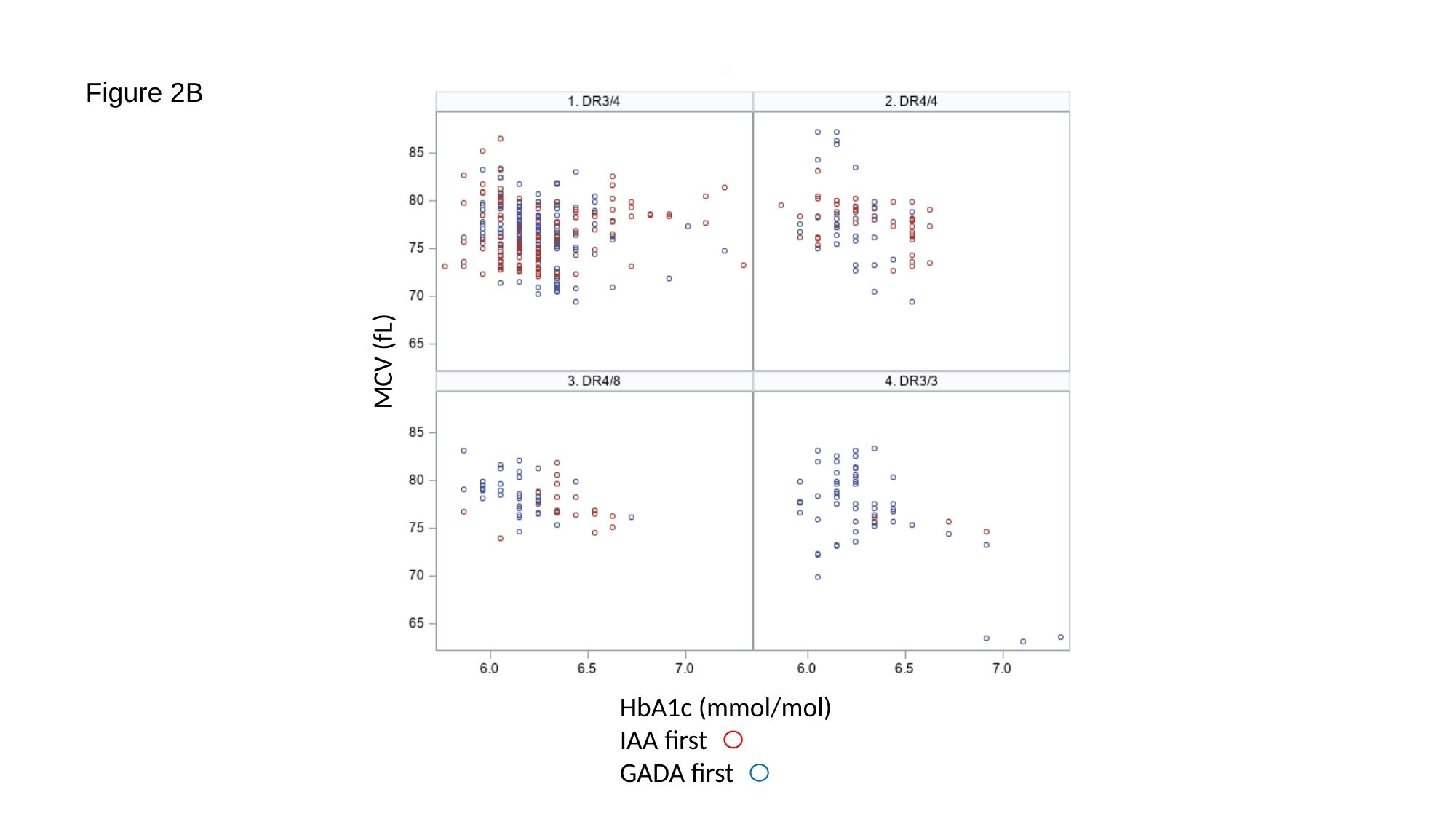

Figure 2B
MCV (fL)
HbA1c (mmol/mol)
IAA first
GADA first
