# Supplementary material for: Complete blood counts with red blood cell determinants associate with reduced beta‐cell function in seroconverted Swedish TEDDY children
Source: Endocrinol Diabetes Metab. 2021 May 3;4(3):e00251. doi: 10.1002/edm2.251 (PMC8279594; doi:10.1002/edm2.251)
Supplement: Supplementary file 2 — Supplementary Material [file EDM2-4-e00251-s002.docx]

**Electronic Supplementary Material**

Supplementary Table 1. Levels of HbA1c and OGTT measures with the number of islet β-cell autoantibody positive children in the study population being followed for the respective measure type.

| Measurement at first observation | Single IAA | Multiple IAA |
| --- | --- | --- |
| HbA1c  n  Median (%, mmol/mol) (SD)  Min - Max | 30  6.2 (0.3)  5.9 - 7.0 | 49  6.2 (0.2)  5.9 - 6.7 |
| OGTT |  |  |
| Fasting Glucose |  |  |
| N |  | 47 |
| Median (mg/dL) (SD) |  | 85.0 (88.8) |
| Min – Max |  | 60 - 114 |
|  |  |  |
| Glucose AUC  Time point 0 and 120 min |  |  |
| N |  | 39 |
| Median (SD) |  | 102 (25) |
| Min – Max |  | 66-211 |
|  |  |  |
| Fasting Insulin |  |  |
| N |  | 40 |
| Median (SD) (uU/mL) |  | 3.7 (2.4) |
| Min - Max |  | 1.1 – 13.1 |
|  |  |  |
| Fasting C-peptide |  |  |
| n |  | 42 |
| Median (SD) (ng/L) |  | 0.8 (0.4) |
| Min – Max |  | 0.2 – 2.3 |
|  |  |  |

**Supplemental figure** **legend**

Figure 1. Predicted trajectories from the mixed models analysis for Lymphocytes, Monocytes (**A**), Red blood cells, Hemoglobin, MCH, and MCV (**B**), stratified by the number of islet autoantibodies (single or multiple ≥ 2 islet autoantibodies) at initial observation.  Each trajectory represents a single subject.

Figure 2 **A**. Mean corpuscular hemoglobin (MCH) in relation to HLA category (DR3/4, DR4/4, DR4/8 and DR3/3) in children who developed insulin autoantibodies (IAA) as the first autoantibody or glutamic acid decarboxylase autoantibodies (GADA) first.

**B**. Mean corpuscular volume (MCV) in relation to HLA category (DR3/4, DR4/4, DR4/8 and DR3/3) in children who developed insulin autoantibodies (IAA) as the first autoantibody or glutamic acid decarboxylase autoantibodies (GADA) first.

**The TEDDY Study Group**

**Colorado Clinical Center:** Marian Rewers, M.D., Ph.D., PI^1,4,5,6,9,10^, Aaron Barbour, Kimberly Bautista^11^, Judith Baxter^8,911^, Daniel Felipe-Morales, Kimberly Driscoll, Ph.D.^8^, Brigitte I. Frohnert, M.D.^2,13^, Marisa Stahl, M.D.^12^, Patricia Gesualdo^2,6,11,13^, Michelle Hoffman^11,12,13^, Rachel Karban^11^, Edwin Liu, M.D.^12^, Jill Norris, Ph.D.^2,3,11^, Stesha Peacock, Hanan Shorrosh, Andrea Steck, M.D.^3,13^, Megan Stern, Erica Villegas^2^, Kathleen Waugh^6,7,11^. University of Colorado, Anschutz Medical Campus, Barbara Davis Center for Childhood Diabetes.

**Finland Clinical Center:** Jorma Toppari, M.D., Ph.D., PI^¥^1,4,10,13^, Olli G. Simell, M.D., Ph.D., Annika Adamsson, Ph.D.^^11^, Sanna-Mari Aaltonen^^^, Suvi Ahonen*^±§^, Mari Åkerlund*^±§^, Leena Hakola*^±^, Anne Hekkala, M.D.^µ¤^, Henna Holappa^µ¤^, Heikki Hyöty, M.D., Ph.D.*^±6^, Anni Ikonen^µ¤^, Jorma Ilonen, M.D., Ph.D.^¥¶3^, Sinikka Jäminki*^±^, Sanna Jokipuu^^^, Leena Karlsson^^^, Jukka Kero M.D., Ph.D.^¥^3, 13^, Miia Kähönen^µ¤11,13^, Mikael Knip, M.D., Ph.D.*^±5^, Minna-Liisa Koivikko^µ¤^, Merja Koskinen*^±^, Mirva Koreasalo*^±§2^, Kalle Kurppa, M.D., Ph.D.*^±12^, Jarita Kytölä*^±^, Jutta Laiho, Ph.D.*^6^, Tiina Latva-aho^µ¤^, Laura Leppänen^^^, Katri Lindfors, Ph.D.*^12^, Maria Lönnrot, M.D., Ph.D.*^±6^, Elina Mäntymäki^^^, Markus Mattila*^±^, Maija Miettinen^§2^, Katja Multasuo^µ¤^, Teija Mykkänen^µ¤^, Tiina Niininen^±^*^11^, Sari Niinistö^§2^, Mia Nyblom*^±^, Sami Oikarinen, Ph.D.*^±6^, Paula Ollikainen^µ¤^ , Zhian Othmani^¥^, Sirpa Pohjola ^µ¤^, Jenna Rautanen^±§^, Anne Riikonen*^±§2^, Miia Pekkola^*±^, Minna Romo^^^, Satu Simell, M.D., Ph.D.^¥12^, Aino Stenius^µ¤11^, Päivi Tossavainen, M.D.^µ¤^, Mari Vähä-Mäkilä^¥^, Eeva Varjonen^^11^, Riitta Veijola, M.D., Ph.D.^µ¤13^, Irene Viinikangas^µ¤^, Suvi M. Virtanen, M.D., Ph.D.*^±§2^. ^¥^University of Turku, *Tampere University, ^µ^University of Oulu, ^^^Turku University Hospital, Hospital District of Southwest Finland, ^±^Tampere University Hospital, ^¤^Oulu University Hospital, §Finnish Institute for Health and Welfare, Finland, ^¶^University of Kuopio.

**Georgia/Florida Clinical Center:** Jin-Xiong She, Ph.D., PI^1,3,4,10^, Desmond Schatz, M.D.*^4,5,7,8^, Diane Hopkins^11^, Leigh Steed^11,12,13^, Jennifer Bryant^11^, Katherine Silvis^2^, Michael Haller, M.D.*^13^, Melissa Gardiner^11^, Richard McIndoe, Ph.D., Ashok Sharma, Stephen W. Anderson, M.D.^^^, Laura Jacobsen, M.D.*^13^, John Marks, DHSc.*^11,13^, P.D. Towe*. Center for Biotechnology and Genomic Medicine, Augusta University. *University of Florida, Pediatric Endocrinology. ^^^Pediatric Endocrine Associates, Atlanta.

**Germany Clinical Center:** Anette G. Ziegler, M.D., PI^1,3,4,10^, Ezio Bonifacio Ph.D.*^5^, Cigdem Gezginci, Anja Heublein, Eva Hohoff^¥2^, Sandra Hummel, Ph.D.^2^, Annette Knopff^7^, Charlotte Koch, Sibylle Koletzko, M.D.^¶12^, Claudia Ramminger^11^, Roswith Roth, Ph.D.^8^, Jennifer Schmidt, Marlon Scholz, Joanna Stock^8,11,13^, Katharina Warncke, M.D.^13^, Lorena Wendel, Christiane Winkler, Ph.D.^2,11^. Forschergruppe Diabetes e.V. and Institute of Diabetes Research, Helmholtz Zentrum München, Forschergruppe Diabetes, and Klinikum rechts der Isar, Technische Universität München. *Center for Regenerative Therapies, TU Dresden, ^¶^Dr. von Hauner Children’s Hospital, Department of Gastroenterology, Ludwig Maximillians University Munich, ^¥^University of Bonn, Department of Nutritional Epidemiology.

**Sweden Clinical Center:** Åke Lernmark, Ph.D., PI^1,3,4,5,6,8,9,10^, Daniel Agardh, M.D., Ph.D.^6,12^, Carin Andrén Aronsson, Ph.D.^2,11,12^, Maria Ask, Rasmus Bennet, Corrado Cilio, Ph.D., M.D.^5,6^, Susanne Dahlberg, Emelie Ericson-Hallström, Annika Björne Fors, Lina Fransson, Thomas Gard, Monika Hansen, Susanne Hyberg, Fredrik Johansen, Berglind Jonsdottir, M.D., Ph.D.^11^, Helena Elding Larsson, M.D., Ph.D.^6,13^, Marielle Lindström, Markus Lundgren, M.D., Ph.D.^13^, Marlena Maziarz, Ph.D., Maria Månsson Martinez, Jessica Melin^11^, Zeliha Mestan, Caroline Nilsson, Kobra Rahmati, Anita Ramelius, Falastin Salami, Anette Sjöberg, Birgitta Sjöberg, Carina Törn, Ph.D.^3^, Ulrika Ulvenhag, Terese Wiktorsson, Åsa Wimar^13^. Lund University.

**Washington Clinical Center:** William A. Hagopian, M.D., Ph.D., PI^1,3,4,5,6,7,10,12,13^, Michael Killian^6,7,11,12^, Claire Cowen Crouch^11,13^, Jennifer Skidmore^2^, Christian Chamberlain, Brelon Fairman, Arlene Meyer, Jocelyn Meyer, Denise Mulenga^11^, Nole Powell, Jared Radtke, Shreya Roy, Davey Schmitt, Sarah Zink. Pacific Northwest Research Institute.

**Pennsylvania Satellite Center:** Dorothy Becker, M.D., Margaret Franciscus, MaryEllen Dalmagro-Elias Smith^2^, Ashi Daftary, M.D., Mary Beth Klein, Chrystal Yates. Children’s Hospital of Pittsburgh of UPMC.

**Data Coordinating Center:** Jeffrey P. Krischer, Ph.D.,PI^1,4,5,9,10^, Rajesh Adusumali, Sarah Austin-Gonzalez, Maryouri Avendano, Sandra Baethke, Brant Burkhardt, Ph.D.^5,6^, Martha Butterworth^2^, Nicholas Cadigan, Joanna Clasen, Kevin Counts, Christopher Eberhard, Steven Fiske^8^, Laura Gandolfo, Jennifer Garmeson, Veena Gowda, Belinda Hsiao, Christina Karges, Qian Li, Ph.D.^2,3^, Shu Liu, Xiang Liu, Ph.D.^2,3,8,13^, Kristian Lynch, Ph.D. ^5,6,8^, Jamie Malloy, Cristina McCarthy^11^, Jose Moreno, Hemang Parikh, Ph.D.^3^, Cassandra Remedios, Chris Shaffer, Susan Smith^11^, Noah Sulman, Ph.D., Roy Tamura, Ph.D.^1,2,11,12,13^, Dena Tewey, Michael Toth, Ulla Uusitalo, Ph.D.^2^, Kendra Vehik, Ph.D.^4,5,6,8,13^, Ponni Vijayakandipan, Melissa Wroble, Jimin Yang, Ph.D., R.D.^2^, Kenneth Young, Ph.D. *Past staff: Michael Abbondondolo, Lori Ballard, Rasheedah Brown, David Cuthbertson, Stephen Dankyi, David Hadley, Ph.D., Kathleen Heyman, Francisco Perez Laras, Hye-Seung Lee, Ph.D., Colleen Maguire, Wendy McLeod, Aubrie Merrell, Steven Meulemans, Ryan Quigley, Laura Smith, Ph.D.^8,11^.* University of South Florida.

**Project scientist:** Beena Akolkar, Ph.D.^1,3,4,5,6,7,9,10^. National Institutes of Diabetes and Digestive and Kidney Diseases.

**Autoantibody Reference Laboratories:** Liping Yu, M.D.^^5^, Dongmei Miao, M.D.^^^, Polly Bingley, M.D., FRCP*^5^, Alistair Williams*, Kyla Chandler*, Ilana Kelland*, Yassin Ben Khoud*, Huma Zahid*, Matthew Randell *. ^^^Barbara Davis Center for Childhood Diabetes, University of Colorado Denver, *Bristol Medical School, University of Bristol, UK.

**HbA1c Laboratory:** Randie R. Little, Ph.D., Curt Rohlfing. Diabetes Diagnostic Laboratory, Dept. of Pathology, University of Missouri School of Medicine.

**HLA Reference Laboratory:** William Hagopian^3^, MD, PhD, Christian Chamberlain, Jared Radtke, Sarah Zink. Pacific Northwest Research Institute, Seattle WA. (Previously Henry Erlich, Ph.D.^3^, Steven J. Mack, Ph.D., Anna Lisa Fear. Center for Genetics, Children’s Hospital Oakland Research Institute.)

**OGTT Laboratory:** Santica M. Marcovina, Ph.D., Sc.D., Andrew N. Hoofnagle, M.D., Ph.D., Northwest Lipid Metabolism and Diabetes Research Laboratories, University of Washington.

**Repository:** Sandra Ke, Niveen Mulholland, Ph.D. NIDDK Biosample Repository at Fisher BioServices.

**Other contributors:** Thomas Briese, Ph.D.^6^, Columbia University. Suzanne Bennett Johnson, Ph.D.^8,11^, Florida State University. Eric Triplett, Ph.D.^6^, University of Florida.

***Committees:***

^1^Ancillary Studies, ^2^Diet, ^3^Genetics, ^4^Human Subjects/Publicity/Publications, ^5^Immune Markers, ^6^Infectious Agents, ^7^Laboratory Implementation, ^8^Psychosocial, ^9^Quality Assurance, ^10^Steering, ^11^Study Coordinators, ^12^Celiac Disease, ^13^Clinical Implementation.
